# Two Different Types of Sarcoma Occurring Synchronously: The Impact of Molecular Biology on Therapeutic Strategy

**DOI:** 10.7759/cureus.21626

**Published:** 2022-01-26

**Authors:** Rabih Mikhael, Giovanni B Damiani, Toufik Bouhadiba, Dimitri Tzanis, Sylvie Bonvalot

**Affiliations:** 1 Department of Surgery, Institut Curie Flag Trouillet-Rossignol, Paris, FRA

**Keywords:** well differentiated sarcoma, pleomorphic sarcoma, synchronous sarcomas, sarcoma surgery, two primary sarcomas, limb sarcoma, retroperitoneal sarcoma

## Abstract

Sarcoma is a rare type of tumor that can arise in the different types of connective tissues. Symptoms vary depending on the size, type, and location of the tumor. Management and surgery should be performed in a referral sarcoma center with a molecular biology platform and a dedicated medical staff. A preoperative percutaneous core needle biopsy (CNB) is required to tailor the medical and surgical strategies. In this report, we describe the case of a well-differentiated retroperitoneal liposarcoma (WD LPS) discovered in the context of recurrent cystitis on an abdominal CT scan, synchronously occurring, with another different type of sarcoma, undifferentiated pleomorphic sarcoma (UPS) developing in the thigh, discovered because of sciatic pain. This extremely rare condition was confirmed by the molecular analysis and justifies a specific strategy taking account of the differential risk. This event should also encourage genetic counseling.

## Introduction

One-third of sarcomas occur in the trunk, whereas 50% occurs in the extremities with more than 150 subtypes according to WHO classification [1,2]. Symptoms are usually nonspecific, such as pain, palpation of a lump, an increase of abdominal girth, and gastrointestinal obstruction by the mass effect. Core needle biopsy (CNB) is the standard of care to adapt treatment strategy. CNB has a high degree of accuracy in the diagnosis of soft-tissue tumors, and it avoids the complications of open biopsy. Surgical management is a “one bloc” excision with clear margins [3,4]. We describe a rare case of two different types of sarcoma, the first sarcoma is a right-sided well-differentiated retroperitoneal liposarcoma (WD LPS), and the other sarcoma, synchronously occurring, left-sided posterior thigh undifferentiated pleomorphic sarcoma (UPS), requiring a specific management.

## Case presentation

**Figure 1:**
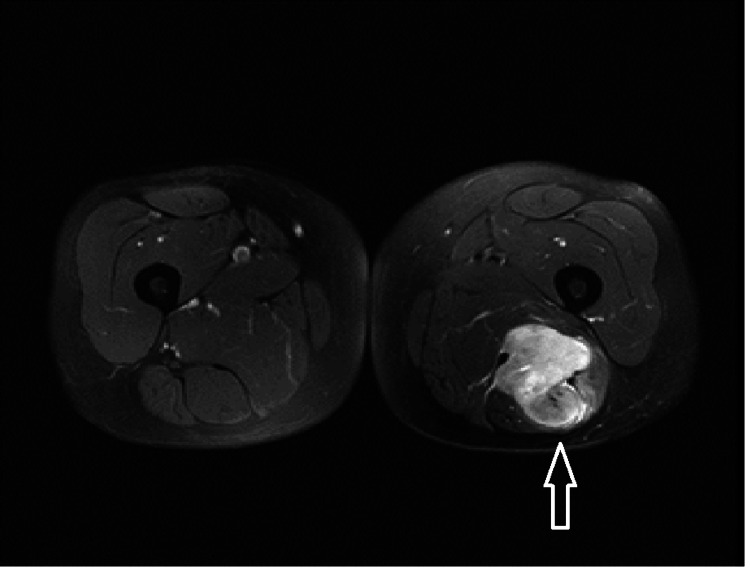
MRI of the thighs showing a heterogeneous tumor (arrow) of the thigh encasing partially the sciatic nerve, measuring 6*6 cm

**Figure 2:**
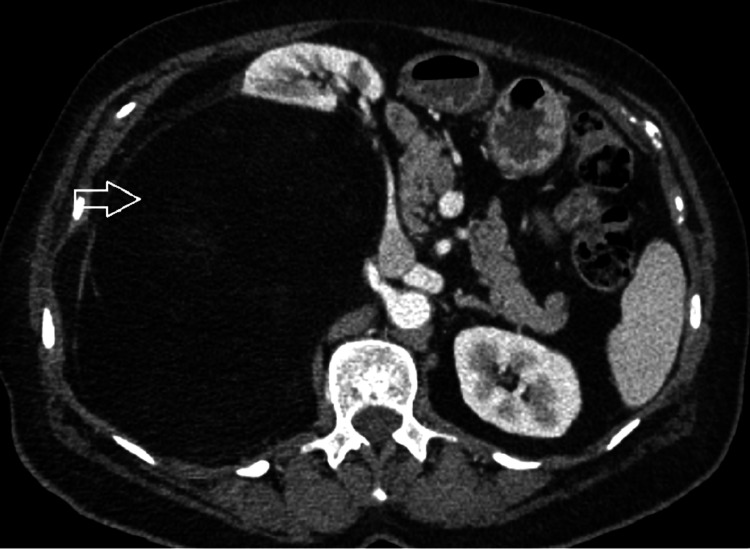
Abdominal CT scan with iv contrast showing a right retroperitoneal mass (white arrow) displacing the right kidney and the right colon anteriorly CT scan showing the retroperitoneal liposarcoma.

**Figure 3:**
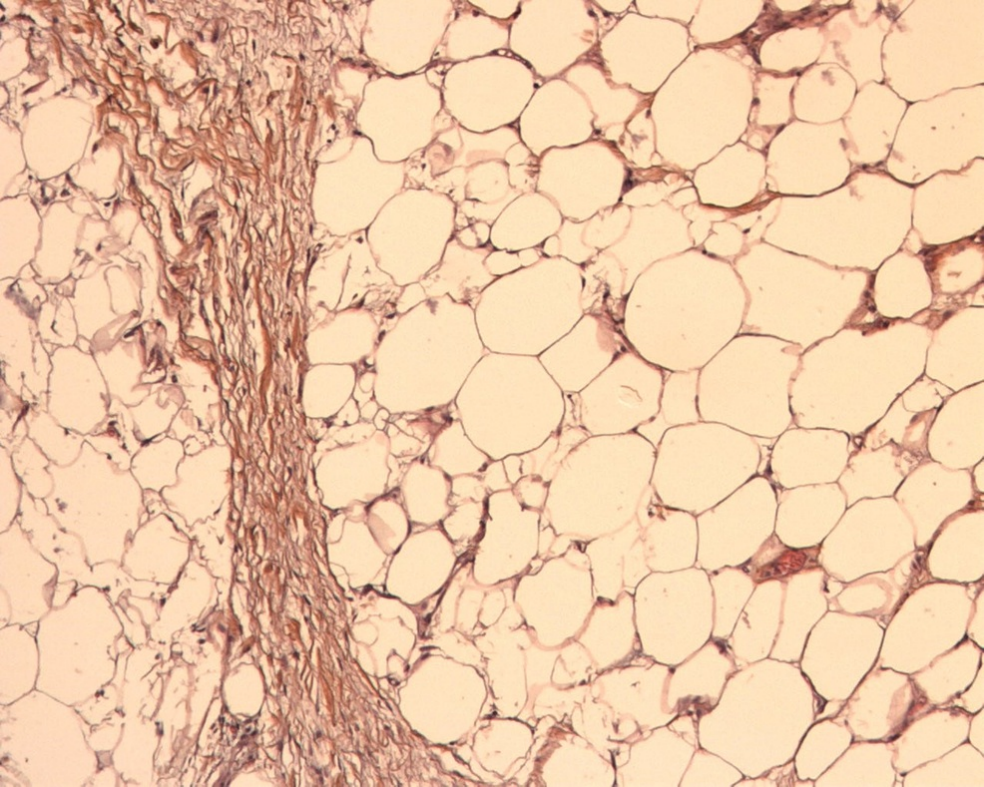
Well-differentiated liposarcoma (high power field [HPF], × 100) Histological examination revealed multivacuolated lipoblasts, with a striking variation in adipocytic size. Fat lobules are admixed with bands of fibrotic stroma containing spindle cells with enlarged hyperchromatic nuclei.

**Figure 4:**
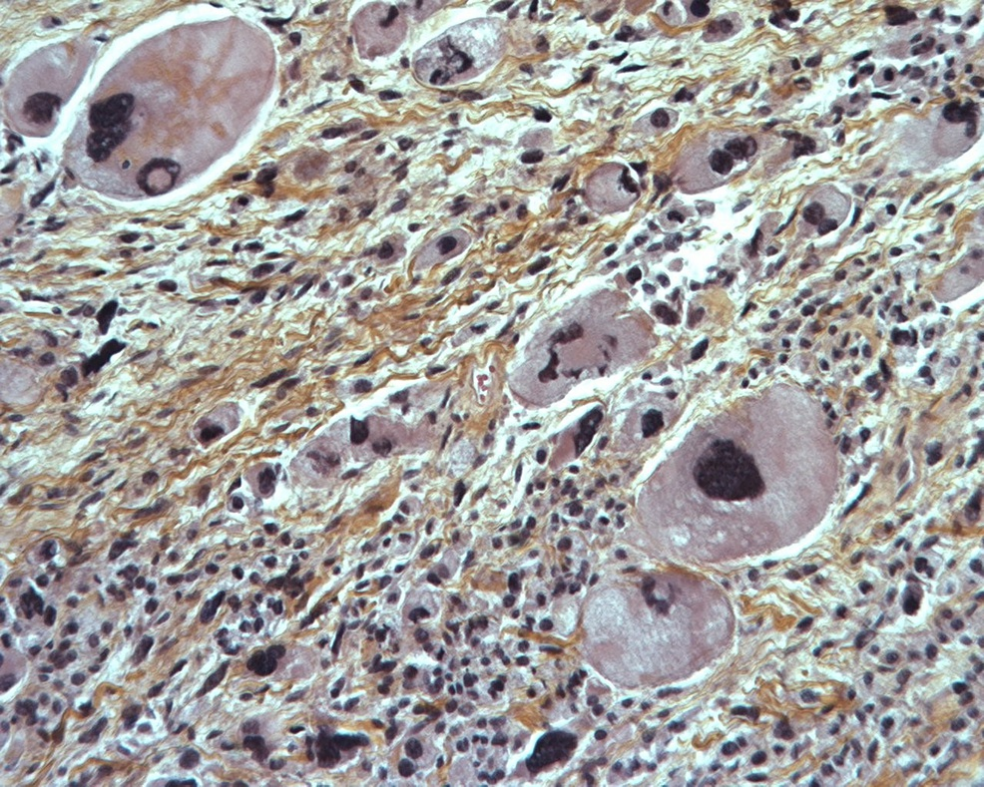
Undifferentiated pleomorphic sarcoma (high power field [HPF], × 400) Histology revealed characteristic spindle and epithelioid cells with marked pleomorphism. A striking variability in nuclear size is noted. Mitotic figures are numerous.

**Figure 5:**
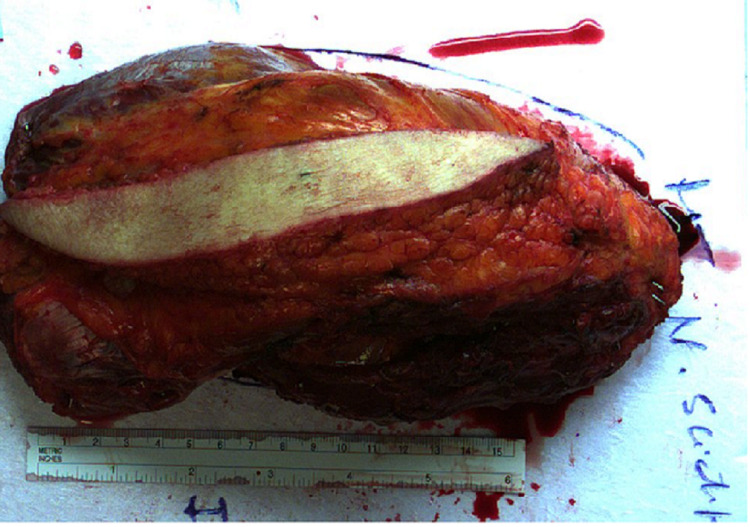
“En-bloc” resection of the thigh tumor "En bloc" resection of the pleomorphic sarcoma with a rim of normal muscle, subcutaneous fat, skin, muscles, and nerve (not seen in this picture). The minimum clear margin was 8 mm.

**Figure 6:**
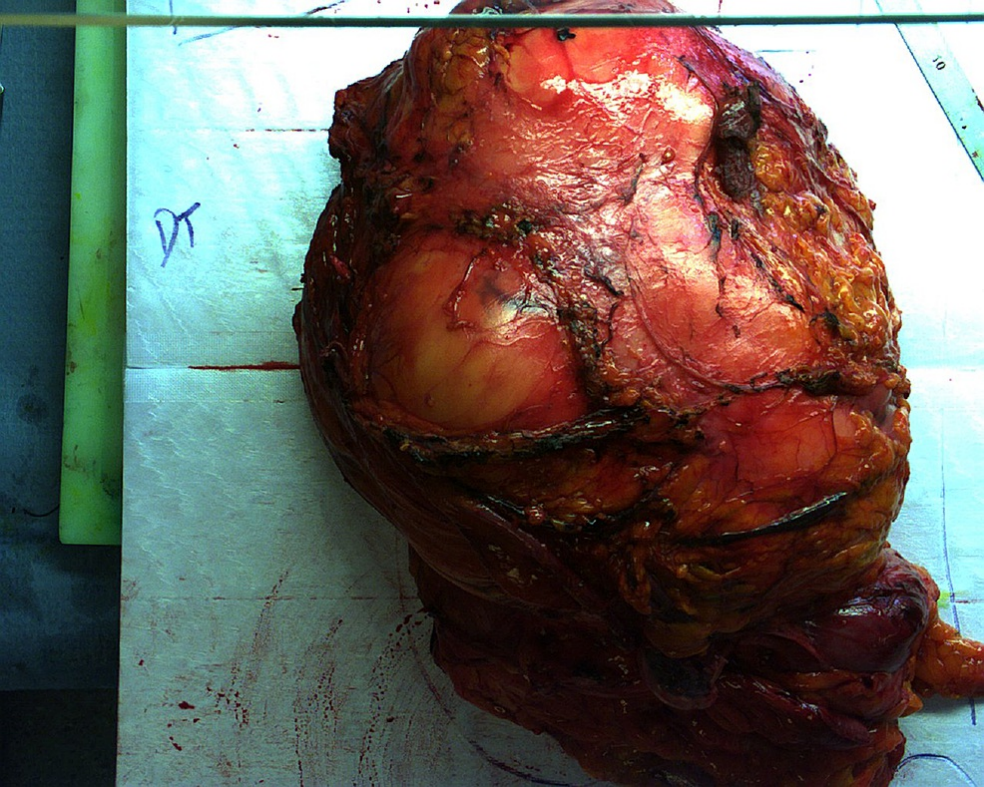
Compartmental resection of the retroperitoneal LPS Complete resection in one bloc of the tumor with the right colon and the right kidney in front, the right adrenal gland inside, the aponeurosis of the iliopsoas in the back. Measuring 26 cm. LPS - liposarcoma

The patient is an 80-year-old female, hypertensive, dyslipidemic with no prior history of cancer or sarcomas. Her brother was diagnosed at the age of 73 with lung (bronchial) cancer due to smoking, and the other brother was diagnosed with colon cancer at the age of 73. The patient had recurrent cystitis for which her treating general practitioner (GP) prescribed an abdominal CT scan that showed a right retroperitoneal adipocytic tumor of 11 cm in the anterior-posterior axis and 10 cm in the transverse axis, encasing the right kidney partially. Without any multidisciplinary tumor board (MTB), the initial strategy conducted by her GP was surveillance. After one year, the patient suffered from a left thigh pain that motivated her GP to prescribe a thigh MRI that showed a heterogeneous tumor of the posterior left thigh (Figure 1) of 6 cm in the anteroposterior (AP) axis and 6 cm in the transverse axis with direct contact with the sciatic nerve. This is when the patient was transferred to our referral sarcoma unit, where a new total abdominal and pelvic CT scan was done and showed an increase in the size of the retroperitoneal adipocytic tumor, measuring 17 cm in the transverse axis and 16 cm in the AP axis (Figure 2). Both tumors were biopsied according to guidelines [3,4]. The retroperitoneal tumor was a WD LPS (Figure 3) grade 1 with amplification of murine double minute 2 (MDM2) on the FISH (fluorescence in situ hybridization), whereas the left thigh tumor was a different type of sarcoma, a grade III UPS (Figure 4) with no amplification of MDM2 on the FISH. After the MTB, it was decided to treat the thigh tumor first because of its high grade and, therefore, the high risk of local progression and metastasis. It was decided to start with surgery and to deliver radiotherapy postoperatively. A compartmental surgical resection of the tumor with its surrounding muscles (semi-tendinous muscle, biceps femoris, and the semi membranous muscle) was performed (Figure 5), followed by 50 Gy radiation therapy. The postoperative period was uneventful. Histopathology reported a 6x6 cm UPS with free margins. One month after the end of the radiation therapy, a new abdominal-pelvic CT scan was performed and showed an increase in the size of the right-sided retroperitoneal liposarcoma (LPS) measuring 21 cm in the AP axis and 17 cm in the transverse one, compressing the inferior vena cava. Based on these findings the MTB decided to treat the retroperitoneal WD LPS surgically. The surgery was a compartmental resection of the tumor with the ipsilateral right colon, kidney, adrenal gland, ovary, and psoas aponeurosis (Figure 6). An omentoplasty was also done with an ileo-transverse bowel anastomosis. Histopathology reported a 21 cm WD LPS. No further treatment was delivered for the retroperitoneal sarcoma. The postoperative course was uneventful.

## Discussion

Sarcomas are rare tumors [1]; however, two different types, synchronously occurring sarcomas, at two different sites in the body, as proved by molecular biology, is an exceptional event justifying a specific therapeutic strategy. Well-differentiated liposarcoma (WD LPS) is a low-grade tumor composed of proliferating mature adipocytes [2]. This is a simple genetic sarcoma characterized by an amplification of the MDM2 gene [2]. They do not metastasize but have a high propensity for local recurrence [5]. Undifferentiated pleomorphic sarcoma (UPS), a different type of sarcoma, is high-grade sarcoma with complex genetic and accounts for less than 5% of LPS [2]. It is identified by a lack of specific immunohistochemical markers for a specific lineage of differentiation and multiple various molecular anomalies. These UPS are very aggressive, and they rapidly enlarge with a high risk of metastasis [5]. These two different types of sarcoma arise in elderly patients (median age around 60 years). Management of these tumors should be conducted in a specialized center with dedicated surgeons [6]. After adapted imaging including an assessment of extension, the standard of care is to perform a pre-operative CNB to adapt the medical and surgical strategy in the MTB [3,4]. In our case, MDM2 amplification by FISH was useful to differentiate WD LPS vs. other benign lipomatous tumors that can grow in the retroperitoneum [7]. The fact that influenced the therapeutic strategy was that the thigh sarcoma was MDM2 negative on immune histochemistry (IHC) and molecular biology proved that the two sarcomas (the thigh sarcoma and the retroperitoneal sarcoma) were two different types of sarcomas and that one was not the metastasis of the other one, which could have led to a non-surgical treatment [3]. The difference in the degree of differentiation of these two different sarcomas helped us decide to treat the thigh sarcoma first, which had a higher risk of progression. The mainstay of the surgical treatment of sarcoma is wide excision with free margins [3,4]. This was achieved in the thigh by the planned resection of the tumor with the adjacent muscles and fascia. In retroperitoneal sarcomas, en bloc resection with the infiltrated and/or adherent organs is recommended, particularly in LPS [4,8]. In the limbs, radiotherapy is validated by two randomized studies in extremities in high-risk sarcomas [9]. Conversely, in retroperitoneal sarcoma (RPS), pre-operative radiotherapy is not a standard of care with a recent negative randomized study (STRASS) [10]. Radiotherapy of the thigh was delivered postoperatively. Indeed, oncological outcomes are the same after pre or post-operative radiotherapy, but pre-operative radiotherapy exposes the limb to more wound complications, especially when the location of the tumor is on the internal side of the thigh and when the skin is fragile in elderly patients [11]. Adjuvant chemotherapy is not a standard of care, especially in an elderly patient [3]. Finally, in front of two different sarcomas occurring in the same patient, oncogenetic counseling would have been useful to look for pathogenic variations in cancer genes [12]. Indeed, this could have an impact on the surveillance strategy and implications in the family screening. However, our patient refused these analyzes.

## Conclusions

The occurrence of simultaneous, different histologic types of sarcoma is extremely rare. Percutaneous biopsy is mandatory to tailor the treatment strategy. Biomolecular and histologic analyses are required to differentiate each tumor’s type. The grade and histological subtype guide further management. Well-differentiated retroperitoneal sarcoma does not metastasize but progresses slowly and locally, whereas the undifferentiated pleomorphic sarcoma of the thigh is at risk of local and metastatic progression. This is the reason why the tumor with the highest potential for metastasis was treated first. Adjuvant radiotherapy is the standard of care for high-grade sarcoma of the extremities, validated by two randomized studies. A recent randomized study (STRASS) did not show a significant benefit in terms of local control for RPS, although a signal of activity has been shown in LPS. Complete, en bloc, gross resection is the cornerstone of management of RPS with the objective to achieve macroscopically complete resection, with a single specimen encompassing the tumor and involved contiguous organs. Given the uncertainty regarding margins definition, a compartmental approach with the resection of adherent visceral irrespective of expected microscopic infiltration should be considered for retroperitoneal LPS. In front of two different sarcomas affecting the same patient, genetic counseling is advised.
